# Clinical characteristics of simple bone cyst associated within florid cemento-osseous dysplasia: a meta-analysis

**DOI:** 10.4317/medoral.27205

**Published:** 2025-08-16

**Authors:** Léonie Rave, Marie Rollin, André Luís Porporatti, Ihsène Taïhi

**Affiliations:** 1 DDS, Faculty of Dentistry, Health Department, Paris Cité University, Paris, France; 2 Odontology Department, Oral Surgery Section, Rothschild Hospital, AP-HP, Paris, France; 3 Oral Surgery Department, Saint Antoine Hospital, Paris, France; 4 Oral Medicine Department, Pitié Salpêtrière Hospital, AP-HP, Paris, France; 5 PhD, Paris Cité University, INSERM 1333, Oral Health Department, Montrouge, France

## Abstract

**Background:**

The objectives of this study were to better understand the association between simple bone cysts (SBC) diagnosed within florid cemento-osseous dysplasia (FCOD) by systematically reviewing the literature with a meta-analysis. This review allows us to study the epidemiology, characteristics, treatments, and follow-up of the association between these two entities.

**Material and Methods:**

A systematic review was performed on PubMed, Scopus, Cochrane library, Embase and Google Scholar. Articles reporting primary studies, case reports and case series were included. We reviewed all articles between 1976 and November 2024. Recorded data were patient’s characteristics, clinical and radiological features, diagnosis, treatment, and follow-up. The meta-analysis was performed for the prevalence of the localization of SBC associated FCOD and the number of SBC lesions within FCOD.

**Results:**

A total of 615 studies were assessed for eligibility by reading titles and abstracts. Finally, 16 articles were included, with 51 patients presenting SBC associated with FCOD. Patients were mostly women (92%) with most of an African origin (81,25% ). The mean age was 41-year-old. The usual treatment for the cyst was surgical exploration with bleeding stimulation to improve healing. After surgical procedure, a majority of the followed-up cases healed completely (52%) or partially (32%). Eight cases reported a recurrence during the mean follow-up period of 6,6 years.

**Conclusions:**

SBC and FCOD are two conditions which can be rarely associated. This may be under-diagnosed as SBC can be misdiagnosed as part of the FCOD lesions. This review highlights the similar clinical characteristics to FCOD which makes important to find out more clinical and radiographic diagnosis criteria to better diagnose and provide the adequate treatment to obtain bone healing.

** Key words:**Florid cemento-osseous dysplasia, meta-analysis, simple bone cyst, systematic review.

## Introduction

Florid Cemento-Osseous Dysplasia (FCOD) of the jaw is defined as a multifocal dysplastic lesion that is constituted of cellular fibrous connective tissue with bone and cementum-like tissue. It belongs to benign odontogenic lesions of the jaw with a periodontal ligament origin. This condition has a high prevalence in middle-aged and elderly black women (1).

The management of this condition relies mainly on annual check-up visits to assess the evolution of the lesions. A surgical intervention is rarely necessary except in cases of infectious complications as osteomyelitis (1).

FCOD can be associated with other osseous lesions, like aneurysmal cyst, central giant cell granuloma, and in rare cases with a Simple Bone Cyst (SBC). SBC is defined as a non-epithelium lined formation surrounded by bone walls with either no content or a liquid and/or connective tissue content (2). This entity is also called traumatic bone cyst, however, the term “simple bone cyst” is the one chosen by the WHO in the new classification of odontogenic tumors and cysts (3).

Melrose *et al* (4) first reported a case of FCOD associated with a SBC and it was followed by several reports.

The overlap in radiographic features between FCOD and SBC creates substantial diagnostic difficulties, as distinguishing between them often necessitates surgical exploration, which is not performed everytime (4). SBCs are generally characterized by distinct, well-defined radiographic borders, a feature that could help differentiate them from periapical lesions typically seen in FCOD cases (5). However, without established radiological criteria, misdiagnoses can occur, leading to unnecessary interventions such as endodontic treatments or extractions, which might inadvertently trigger new dysplastic lesions in the affected areas (6). This underscores the need for standardized imaging protocols and diagnostic guidelines to improve patient outcomes in cases involving these complex jawbone conditions.

To better understand the coexistence of these two jawbone entities, a systematic review was conducted. To the best of our knowledge, no systematic review or meta-analysis have addressed clinical parameters of FCOD associated with SBC. Therefore, based on this premise, the objective of this meta-analysis was to investigate the following question: “Which are the epidemiologic, demographic and clinical characteristics of SBC lesions in patients with a FCOD?’ We searched for parameters as Ethnics, Gender, Age, Localization and Number of Lesions, Use of Biopsies, Surgical Exploration Radiological Examination and Follow-up (time, recurrences and healing process).

## Material and Methods

- Protocol and registration

This systematic review of the literature was carried out according to the Preferred Reporting Items for Systematic Reviews and Meta Analyses (PRISMA) statement (Page *et al*, 2021) (see Supplement 1). The review was registered in the PROSPERO International Prospective Register of Systematic Reviews (National Institute for Health Research, University of York, Centre for Reviews and Dissemination) under the number CRD42022325597.

- Eligibility criteria

This systematic review was designed to assess the following focused question: “What are the epidemiologic, demographic and clinical characteristics of SBC lesions in patients with a FCOD”. The focus question proposed according to PECOS principles: P) Population: Patients presenting FCOD lesions; E) Exposition: SBC associated lesions; C) Control: No control; O) Outcomes: Ethnics, Age, localization and number of FCOD and SBC lesions, use of biopsies, surgical exploration and radiological examination, follow-up (time, recurrences and healing process); S) Studies: primary studies, case reports and case series. The search strategy was applied without gender or date restrictions. Only studies written in English or French were included. The exclusion criteria encompassed: 1) Studies which presented cases without association between FCOD and SBC; 2) Studies with other form of osseous dysplasia and 3) Literature reviews, intervention studies, letters, and personal opinions.

-Information sources

Detailed individual search strategies were developed, in English, for each bibliographic electronic database: PubMed (including Medline), Scopus, Cochrane library, and Embase. A search for grey literature was carried out using Google Scholar, with all databases reviewed from their initial coverage dates up to November 2024. Additionally, the authors hand-searched the reference lists of the selected articles for any additional references and cases reports that might have been missed in the database searches.

-Search strategy

Keywords and Mesh terms were fully explored based on a complete search strategy with fulfilled the terms and similar: “florid cemento osseous dysplasia” OR “cemento osseous dysplasia” OR “florid osseous dysplasia” OR “fibro-osseous lesions” AND “simple bone cyst” OR “solitary bone cyst” OR “traumatic bone cyst”. More information on the search strategies is provided in Supplement 2. All references were managed, and the duplicated hits were removed by reference manager software (Zotero software, Corporation for Digital Scholarship, Virginia, USA).

-Study selection and data collection process

This section followed a two-step process. First, three authors (L.R., M.R. and I.T.) independently analyzed the titles and abstracts of all identified electronic database citations. Secondly, the same authors examined the full-text documents. At each step, they independently screened the studies, applied the eligibility criteria, extracted essential information, and cross-verified the data to ensure accuracy. In order not to exclude potentially relevant articles, questionable abstracts were also included in the full text analysis. The ultimate selection was determined exclusively through a full-text evaluation of the studies.

For each of the included studies, two authors (M.R and L.R.) collected the following items: sex, mean age, origin, number of SBC lesions, localization of FCOD and SBC lesions, 2D and 3D radiological exploration, surgical exploration, biopsies, and follow-up period, healing, and recurrences. In cases the required data were incomplete, the reviewers reached out to the study authors to obtain any unpublished information. Over a 30-day period, they made three contact attempts via email, addressing the first, second and last author respectively.

- Risk of bias in individual studies

The methodological quality of the included case reports was evaluated through the case report and case assessment tool (7). All reviewers reached a consensus on the scoring criteria prior to initiating the critical appraisal process. The same three reviewers (L.R., M.R. and I.T.) worked out any differences regarding data analysis. Following these answers, the risk of bias was categorized as follows: 1) Low risk of bias, if all criteria were met, 2) Unclear risk of bias, if one or more criteria were not exactly described, and 3) High risk of bias, if one or more criteria were not met. The quality assessment Figures for all included studies were created using Review Manager 5.3 (RevMan 5.3, developed by The Nordic Cochrane Centre, Copenhague, Denmark).

- Study outcomes and summary measures

Any type of outcome measurement was considered. Authors attempted to standardize the measurements in mean and standard deviation (SD) or percentage (%) with 95% confidence intervals (95% CI) when feasible.

- Synthesis of results

A meta-analysis was conducted to statistically pool the data whenever the studies were deemed compatible and sufficiently similar in terms of design, exposures and outcomes. Heterogeneity among studies was assessed either by examining clinical factors (such as differences in participants, types of expositions, and outcomes), methodological aspects (including study design, and risk of bias) and statistical features (study effect). Additionally, the inconsistency index (I2) statistical test was employed to quantify heterogeneity (Higgins, JPT 20).

If quantitative synthesis was appropriate, a proportion analysis was performed with the aid of MedCalc® statistical software (MedCalc software Ldt, Ostend, Belgium) for the univariate analysis. Heterogeneity was assessed using the Cochran Q test and I2 statistics. The choice between a fixed-effect or random-effect model for the analysis depended on the assumption of whether the intervention effects were genuinely uniform. Heterogeneity was measured using the I2 statistic, with values exceeding 50% indicating substantial variability between studies. The threshold for statistical significance was establish at 5%.

Meta-analyses of sub-groups were conducted to assess prevalence of mandibular localization of SBCs and the of unique lesions, with 95% CI.

- Risk of bias across studies

The overall risk of bias across studies was evaluated to determine its potential impact on the meta-analysis results. Both methodological and statistical heterogeneity were assessed by analyzing differences in study design and the presence of bias.

- Confidence in cumulative evidence

The overall strength of the available evidence was summarized using "Grading of Recommendations Assessment, Development and Evaluation" (GRADE) Summary of Findings (SoF) Tables, generated with the GRADEpro software. (Supplement 3)

- Data availability statement

All of the data, material and methods which supporting the results can be found in the article or appendices.

## Results

- Study selection 

The electronic search yielded a total of 615 articles and after duplicates removal 503 articles were screened. 63 were selected after title reading for abstract reading. After abstract reading, 33 articles were assessed for phase-two. After complete reading, 16 articles were included in this systematic review and the meta-analysis and 17 were excluded.

**Figure 1:**
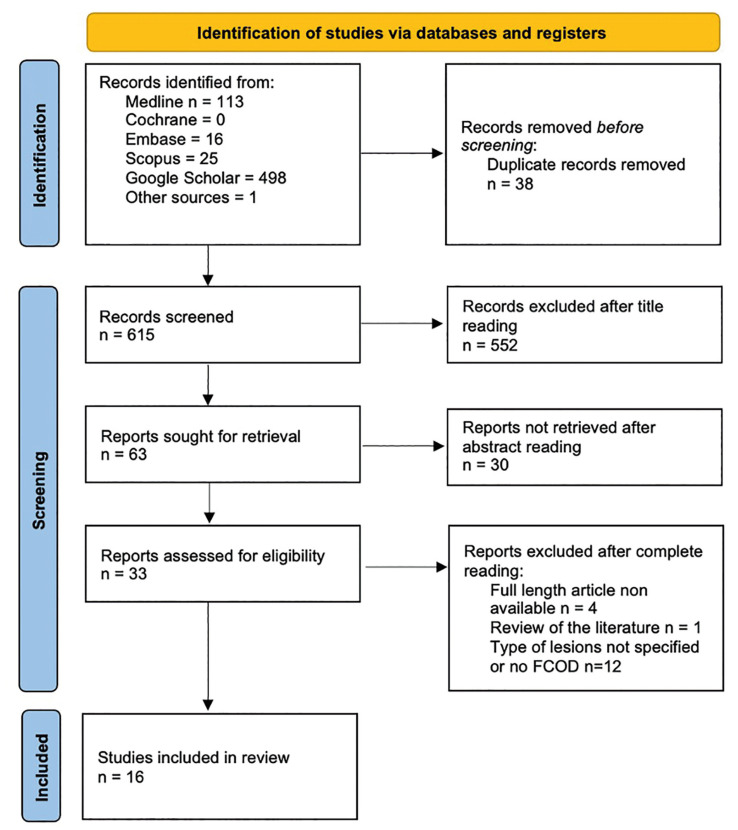
PRISMA Flowchart. From: Page MJ, McKenzie JE, Bossuyt PM, Boutron I, Hoffmann TC, Mulrow CD, *et al*. The PRISMA 2020 statement: an updated guideline for reporting systematic reviews. BMJ 2021;372:n71. doi: 10.1136/bmj.n71

Fig. 1 illustrates a flowchart detailing the process of study identification, inclusion, and exclusion.

- Study characteristics and results of individual studies

The results are summarized in Table 1, Table 2 and Table 3. A total of 51 patients were included, with a mean age of 41 years old. When mentioned, the origin was in most cases African for 81,25% of the cases (4,8-11), and 92% of all patients were female. The other origins found were Indian (8,12), Oriental (13) and Japanese (14). In eight articles, the patients’ origin was not specified (6,15-21). In the reported cases, FCOD lesions were found to preferentially affect the mandible with a 100% rate, including 35,3% of FCOD affecting both mandible and maxilla and no case of exclusively maxillary localization were found. In one study, the total of cysts for three of the patients was not mentioned, however, only two cysts were found in the maxilla among all the reported cases, demonstrating a clear predominance of mandibular localization of SBCs.

Ten patients (19,6%) showed multiple SBCs : 6 patients showed 2 cysts and 4 patients showed 3 cysts or more. 41 patients (80,4%) showed only one SBC. Two patients with multiple cysts showed SBC in the maxilla.

Only half of the studies explored the lesions with 3D radiographs such as CT scan in most cases, MRI and scintigraphy (19). Biopsies were performed in all the studies to confirm FCOD diagnosis, except of two studies (8,20). In total, 38 cases had a confirmed FCOD histological diagnosis (74,45%). Surgical exploration was performed and confirmed the diagnosis of SBC in all the studies except of one study with 8 patients (20). A curettage was performed concomitantly with surgical exploration in 83% of the cases.

Only 25 patients (49,01%) were followed up. Among them, 13 patients (52%) had a complete healing and 8 patients (32%) had a partial healing, after a mean of 2-3 years of follow-up in most studies. This demonstrates that surgical exploration and curettage of the bone wall can improve the healing of the lesion. For the remaining 4 patients (16%), the healing was not mentioned. The period of follow-up varied from 6 months to 29 years.

Among the followed-up cases, a total of 8 cases of recurrence (15,7%) were found, either as recurrence of the initial lesion (3 patients), enlargement of the cavity in cases with partial healing (3 patients), or appearance of new lesions (2 patients). The average time of diagnosis of the recurrence during follow-up is approximately 6,6 years.

- Meta-analysis

**Figure 2:**
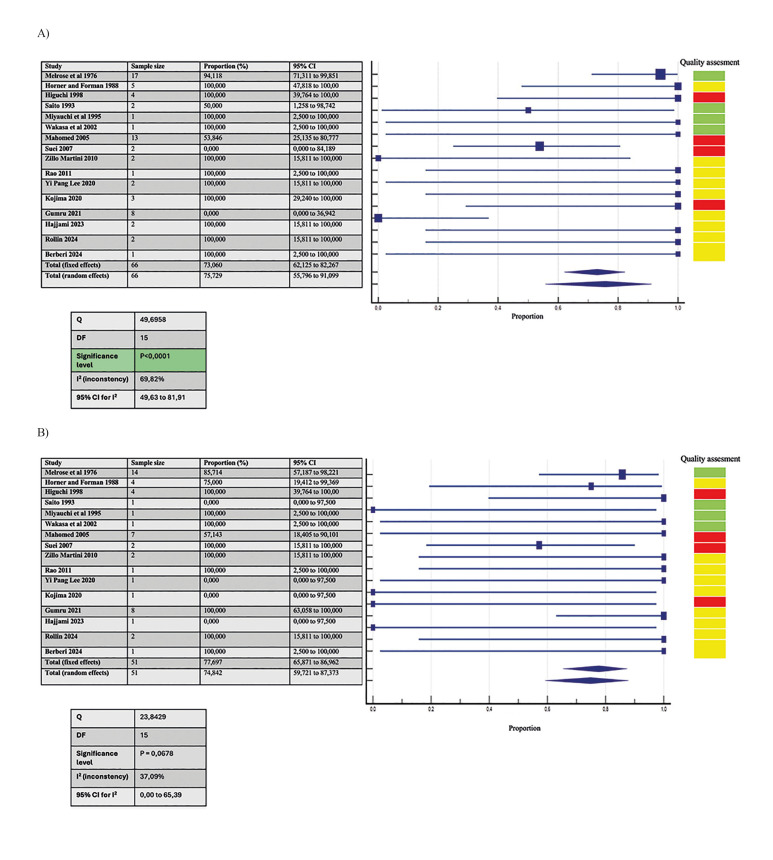
A. Proportion of mandibular SBC lesions in FCOD cases. B. Proportion of patients with only one SBC lesion associated with FCOD cases. On the right, the quality assessment of the articles using the bias table (green=good, yellow=fair, red=poor).

The meta-analysis of the prevalence of mandibular localization of SBC associated to FCOD and the prevalence of single SBC lesions within FCOD was performed for the 16 studies of the systematic review. The prevalence of mandibular localization of the cyst was 75,73% (95% IC 49,63 to 81,91 *n*=66 *p*<0,0001 and high heterogeneity, I2=69,82%). The SBC lesions are found preferentially in the mandible (Fig. 2).

The prevalence of patients presenting a single SBC was 74,84% (95% IC 0 to 65,39 *n*=51 *p*<0,0678 and moderate heterogeneity, I2=37,09%). It seems that patients present mostly only one lesion (Fig. 2).

- Risk of bias in studies

The 16 studies exhibited varying levels of risk of bias. The selected studies had a case report design. The main methodological issue pertained to strategies to deal with confounding factors such as surgical exploration, and follow-up, that were not always collected. We used the case report and case assessment tool (7). The categorization of risk of bias is based on the number of positive responses to the evaluation criteria. A study is considered to have a low risk of bias if most or all answers are positive, typically 7 or 8 out of the total 8 questions. When some responses are negative or unclear, with 4 to 6 positive answers, the risk of bias is classified as uncertain. A high risk of bias is identified when a substantial number of responses are negative or unclear, resulting in fewer than 4 positive answers. The overall assessment may also depend on the relative importance of specific questions, such as those related to the representativeness of the sample or the accuracy of the outcome measurements. Finally, four articles was classified as having a low risk of bias (1,4,22,23), eight as unclear (11,13-16,21,24), and four as high risk of bias (5,8,20,25) (Table 4).

- Confidence in cumulative evidence

The overall quality of evidence, as determined using GRADE’s Summary of Findings Tables, was rated as very low (Supplement 3). This assessment was due to high inconsistency (I2 exceeding 57%), a limited pooled sample size, and the predominance of observational study designs.

## Discussion

Our Study allows to focus on the data concerning the association between FCOD and SBCs which is still rarely described in the literature. The demographic data are in accordance with former findings, showing a large predominance of middle-aged black women. In a recent study published in 2022 by Soluk-Tekkesin *et al* (26), 19 patients with FCOD were analyzed, of whom 16 were female, and all were aged between 20 and 49 years. Similarly, a systematic review conducted by MacDonald-Jankowski *et al* (27) in 2003 reported that 59% of FCOD cases occurred in Black individuals.

Melrose *et al* (4) first described the lesions as florid osseous dysplasia in 1976. Later in 1985, Waldron (1) used the term FCOD because of the presence of both bone and cementum in the lesions. Waldron characterized FCOD (1) as bilateral, symmetrical and densely sclerotic lesions located in the mandibular molar or premolar regions. For SBC, Melrose *et al* (4) first reported 14 cases of solitary bone cyst among 34 patients with FCOD lesions in 1976. This incidence is quite high compared to other studies. The diagnosis of solitary bone cyst could initially be used too often for cavities formed by the FCOD or on the contrary, SBC could be under-diagnosed because of their radiolucent or mixed radiographic aspect mimicking FCOD lesions. It could even be a systematic association between the two entities.

Regarding the clinical presentation, it seems that the localization of the cyst is mostly found in the mandibular regions regardless of the localization of the FCOD lesions. The FCOD lesions could be found in both jaws, but isolated maxillary lesions are non-existent in this review. Concerning radiographic exploration of SBC associated to FCOD, since the beginning 2000s and the development of CT scans, 3D radiological exploration seems to be the gold standard, almost always associated with panoramic radiographs. CT scans allows a better radiographic description of the lesion and can avoid a biopsy to confirm the diagnosis of FCOD lesions. Indeed, FCOD biopsy is almost not recommended regarding the specific radiographic presentation of this entity in most cases. Biopsy may only be considered when disturbing symptoms such as pain, swelling of mucosal lesion are reported. In contrast, the diagnosis of SBC needs a surgical exploration as an empty cavity with bony wall is usually found.

This systematic review allows us to go further in the management of SBC associated with FCOD lesions. It demonstrates that surgical exploration and curettage of the lesion is the proper way to observe a bone healing of the cyst , which was performed in 83% of the cases. Indeed, in most cases, a bone healing is obtained after a mean of 2-3 years of follow-up in most studies. Nevertheless, we reported 8 recurrences that occurred after an average of 6,6 years of follow-up. That indicates that recurrences could be more frequent than previously assumed and may appear years after the initial healing. Long-term follow-up is necessary to detect any possible recurrence.

The meta-analysis conducted on the prevalence of the localization and the number of cysts showed that most lesions are isolated and localized in the mandible. The predominance of mandibular lesions could be explained by different mechanisms. Harnet *et al* (24) suggest that the primary ossification spot of the mandible, near the mental foramen, is the origin of most SBCs because of an abnormality in cellular differentiation during the ossification. Variant mechanisms appear to contribute to the development of SBC in patients with FCOD. One of the most accepted explanations would be that the liquefaction necrosis or resorption of blood clot following intramedullary hemorrhage due to FCOD lesions would result in the destruction of the bone by enzymatic activity, causing the formation of a bone cavity (5). If the FCOD is partly responsible for the formation of the SBC, its mandibular preferential localization could be explained by a general preference for the mandibular localization of SBC and FCOD.

In addition, FCOD has been linked to other bone lesions, such as aneurysmal cysts, as demonstrated by Yeom and Yoon (22) in a case report and review of the literature, as well as glandular odontogenic cysts, with one case was documented by Kungoane (23). In 2017, Sarmento *et al* (25) also reported a case of FCOD associated with a peripheral giant cell granuloma. But these associations remain quite rare.

The dysplastic bone structure in FCOD may predispose these regions to secondary lesions such as SBCs, which are influenced by underlying vascular or cellular abnormalities (15).

Long-term follow-up remains a cornerstone in managing these cases, as recurrence was observed in cases of SBC associated with FCOD. This underscores the necessity of regular radiographic evaluations, ensuring early detection of any recurrence or development of new lesions, and minimizing the risks of complications such as mandibular fractures.

Additionally, the potential for rare associations of FCOD with other lesions like aneurysmal cysts or peripheral giant cell granulomas, as highlighted by Yeom and Yoon (22) and Sarmento *et al* (25), further reinforces the importance of a vigilant and individualized follow-up strategy for patients diagnosed with these conditions. This comprehensive approach would allow for optimized patient outcomes and the prevention of underdiagnosis or overtreatment of associated lesions.

These results are noteworthy but must be interpreted with caution due to several factors that could compromise their validity. Firstly, many of the included studies were retrospective in design, which inherently limits their methodological rigor. Furthermore, essential diagnostic tools such as CBCT and biopsies were not consistently utilized across all studies. Despite this, reliance on characteristic radiological features combined with surgical curettage provides a reasonable degree of confidence in the diagnostic process in most cases. Additionally, some authors did not respond to repeated requests for specific data, resulting in the loss of valuable information.

Underreporting remains a critical issue, as some cases may not have been documented or published due to diagnostic oversight or misclassification, with authors assuming these cases fell within broader categories like FCOD. This underdiagnosis likely skews the available dataset. Moreover, the heterogeneity in follow-up protocols and the limited scope of reported data make it challenging to draw robust conclusions regarding healing rates and recurrence patterns.

Confounding variables, such as surgical exploration methods and follow-up duration, must be meticulously documented and controlled in future studies to enhance reliability and validity.

To address these limitations, prospective, multicentric studies focusing on pre-diagnosed FCOD cases should be prioritized. These studies should incorporate systematic radiological evaluations and surgical explorations to confirm diagnoses and identify associated SBCs. Moreover, the integration of artificial intelligence (AI) tools holds promise for improving diagnostic accuracy by differentiating FCOD from SBCs and other imaging abnormalities (28).

The findings of this review may have limited generalizability due to the inherent constraints of the included studies, potential confounding factors, and the very low GRADE level of evidence. This review highlights the pressing need for improved study designs and more comprehensive clinical data. Future research should emphasize multicentric methodologies to account for cultural and demographic variations, while systematically analyzing confounding factors such as age, sex, surgical exploration techniques, and follow-up protocols to strengthen the evidence base.

## Conclusions

In conclusion, the epidemiologic, demographic, and clinical characteristics of SBC in patients with FCOD show clear trends. Epidemiologically, the mean age of patients is 41 years. Demographically, SBC lesions in this context are predominantly observed in African populations and show a strong female predilection. Clinically, both SBC and FCOD are strongly associated with the mandibular region, with SBCs typically presenting as a single lesion.

The association between FCOD and SBC seems to be quite rare. However, it could be misdiagnosed or under-diagnosed because of the aspect of the lesion that could misthought as part of the FCOD lesion or a cyst of dental origin. For example. It is important to make the diagnosis of SBC when confronted to that type of radiographic image to provide the adequate treatment with surgical exploration and curettage of the bone walls to stimulate the bone healing.

## Figures and Tables

**Table 1:** Demographic information.

Author	Date	Country	Number of cases	Mean Age (yo*)	Origin	Sex
Melrose et al (4)	1976	USA	14	42	African	Female
K. Horner -GH Forman (8)	1988	UK	4	36	3 West Indian	3 Female
					1 West African	1 Male
Higuchi (15)	1988	Japan	4	40,5	Unknown	Female
Saito (16)	1992	Japan	1	42	Unknown	Female
Miyauchi et al (14)	1995	Japan	1	40	Japanese	Female
Wakasa et al (17)	2002	USA	1	34	Unknown	Female
Mahomed (9)	2005	USA	7	42,3	African	6 Female
						1 Male
Suei (6)	2007	Japan	2	Unknown	Unknown	Unknown
Zillo Martini (10)	2010	Brazil	2	54	African american	Female
Rao (12)	2011	Inde	1	42	Indian	Female
Yi Pang Lee (18)	2020	China	1	48	Unknown	Female
Kojima (19)	2020	Japan	1	39	Unknown	Female
Gumru (20)	2021	Turkey	8	47	Unknown	Female
Hajjami (21)	2023	Tunisia	1	31	Unknown	Female
Rollin (11)	2024	France	2	40,5	African	2 Female
Berberi (13)	2024	Lebanon	1	46	Oriental	Female

**Table 2:** Clinical findings.

Author	Number of SBC	Localisation		Radiological exploration	
		SBC	FCOD	2D	3D
Melrose et al (4)	17	1 in the Maxilla (patient with 3 cysts) and 16 in the Mandible	Mandible and Maxilla in 12 patients, only in the Mandible in 2 patients	Yes panoramic and occlusal films	No
	One with 2 cysts				
	One with 3 cysts				
K. Horner -GH Forman (8)	5	Mandible	1 - Maxilla and Mandible	Yes : Panoramic radiographs and occlusal films	No
			2- Mandible and Maxilla		
	One with 2 cysts		3- Mandible		
			4- Mandible and Maxilla (patient with 2 cysts)		
Higuchi (15)	4	Mandible	Mandible	Yes : panoramic radiographs and occlusal films	No
Saito (16)	2	1 in the Mandible	Mandible	Yes : panoramic radiographs and occlusal films	No
	One with 2 cyst	1 in the Maxilla			
Miyauchi et al (14)	1	Mandible	Mandible	Yes : panoramic radiograph and occlusal films	No
Wakasa et al (17)	1	Mandible	Mandible	Yes : panoramic radiographs and occlusal films	Yes : CT scan
Mahomed (9)	1->2	Mandible	6 patients with only Mandible and 1 patient with both Maxilla and Mandible lesions	Yes panoramic radiographs and occlusal films	No
	2->3				
	3->3				
	4 -> 1				
	5->1				
	6->1				
	7->1				
Suei (6)	2	Unknown	Unknown	No	No
Zillo Martini (10)	2	Mandible	Mandible	Yes : panoramic radiographs	Yes : CT scan
Rao (12)	1	Mandible	Mandible and Maxilla	Yes : panoramic radiograph and intra oral X rays	Yes: CT scan
Yi Pang Lee (18)	2	Mandible	Mandible	Yes: panoramic radiograph	Yes : CT scan
Kojima (19)	3	Mandible	Maxilla and Mandible	Yes : panoramic radiographs	Yes : CT scan MRI and Bone scintigram
Gumru (20)	8	Unknown	Mandible only or Mandible and Maxilla number unknown	No	Yes : CT scan
Hajjami (21)	2	Mandible	Mandible	Yes : panoramic radiographs	Yes : CT scan
Rollin (11)	2	Mandible	Mandible	Yes : panoramic radiographs	Yes : CT scan
Berberi (13)	1	Mandible	Mandible	Yes : panoramic radiographs	Yes : CT scan

**Table 3:** Procedures And Follow-Up.

Author	Surgical exploration	Biopsy		Follow-up	Follow-up period	Bone healing	Recurrence
		SBC	FCOD				Time
Melrose et al (4)	Yes	Yes	Yes	9	1 to 29 years	6 Compete3 partial	1. 4 years
							2. 6 years
							3.During 22 years
K. Horner -GH Forman (8)	Yes	Yes	No	3	18 months to 2 years	Complete healing for the cases which were followed-up	No recurrence
Higuchi (15)	Yes	Yes	Yes	No	Unknown	Unknown	Unknown
Saito (16)	Yes	Yes	Yes	1	8 years	Complete	8 years
Miyauchi et al (14)	Yes	Yes	Yes	1	14 months	Partial healing at 12months	14 months
Wakasa et al (17)	Yes	Yes	Yes	1	3 years	Complete bone healing after the second treatment (after recurrence)	3 years
Mahomed (9)	Yes	Yes	Yes	No	Unknown	Unknown	Unknown
Suei (6)	Yes	Yes	Yes	2	3 years	Partial	Between 24 and 29 months
Zillo Martini (10)	Yes	Yes	Yes	1	6 months	Partial	No recurrence
Rao (12)	Yes	Yes	Yes	1	6 months	Unknown	Unknown
Yi Pang Lee (18)	Yes	Yes	Yes	No	Unknown	Unknown	Unknown
Kojima (19)	Yes	Yes	Yes	1	27 months	Gradual regression with bone formation	No
Gumru (20)	Unknown	No	No	No	Unknown	Unknown	Unknown
Hajjami (21)	Yes	Yes	Yes	2	11 years	Partial	No
Rollin (11)	Yes	Yes	Yes	2	9 months to	1 partial	30 month
					30 month	1 complete	
Berberi (13)	Yes	Yes	Yes	No	Unknown	Unknown	Unknown

**Table 4:** Tool for evaluating the risk of bias of case reports and case series (Murad et al, 2018).

Source	Selection	Ascertainment		Causality				Reporting	Total Risk of bias
	Does the patient(s) represent(s) the whole experience of the investigator (center) or is the selection method unclear to the extent that other patients with similar presentation may not have been reported?	Was the exposure adequately ascertained?	Was the outcome adequately ascertained?	Were other alternative causes that may explain the observation ruled out?	Was there a challenge/rechallenge phenomenon?	Was there a dose-response effect?	Was follow-up long enough for outcomes to occur?	Is the case(s) described with sufficient details to allow other investigators to replicate the research or to allow practitioners make inferences related to their own practice?	
Melrose et al (4)	Yes	Yes	Yes	not relevant	not relevant	not relevant	Yes	Yes	Low
K. Horner -GH Forman (8)	Yes	Yes	Yes	not relevant	not relevant	not relevant	Yes	No	Unclear
Higuchi (15)	Yes	Yes	Yes	not relevant	not relevant	not relevant	No	No	High
Saito (16)	Yes	Yes	Yes	not relevant	not relevant	not relevant	Yes	Yes	Low
Miyauchi et al (14)	Yes	Yes	Yes	not relevant	not relevant	not relevant	Yes	Yes	Low
Wakasa et al (17)	Yes	Yes	Yes	not relevant	not relevant	not relevant	Yes	Yes	Low
Mahomed (9)	Yes	Yes	Yes	not relevant	not relevant	not relevant	No	No	High
Suei (6)	Yes	Yes	No	not relevant	not relevant	not relevant	No	No	High
Zillo Martini (10)	Yes	Yes	Yes	not relevant	not relevant	not relevant	No	Yes	Unclear
Rao (12)	Yes	Yes	Yes	not relevant	not relevant	not relevant	No	Yes	Unclear
Yi Pang Lee (18)	Yes	Yes	Yes	not relevant	not relevant	not relevant	No	Yes	Unclear
Kojima (19)	Yes	Yes	Yes	not relevant	not relevant	not relevant	Yes	Yes	Unclear
Gumru (20)	Yes	Yes	Yes	not relevant	not relevant	not relevant	No	No	High
Hajjami (21)	Yes	Yes	Yes	not relevant	not relevant	not relevant	Yes	Yes	Unclear
Rollin (11)	Yes	Yes	Yes	not relevant	not relevant	not relevant	No	Yes	Unclear
Berberi (13)	Yes	Yes	Yes	not relevant	not relevant	not relevant	No	Yes	Unclear

## References

[1] Waldron CA (1985). Fibro-osseous lesions of the jaws. J Oral Maxillofac Surg.

[2] Kumar ND, Sherubin JE, Raman U, Shettar S (2011). Solitary bone cyst. Indian J Dent Res.

[3] Speight PM, Takata T (2018). New tumour entities in the 4th edition of the World Health Organization Classification of Head and Neck tumours: odontogenic and maxillofacial bone tumours. Virchows Arch.

[4] Melrose RJ, Abrams AM, Mills BG (1976). Florid osseous dysplasia. A clinical-pathologic study of thirty-four cases. Oral Surg Oral Med Oral Pathol.

[5] Chadwick JW, Alsufyani NA, Lam EWN (2011). Clinical and radiographic features of solitary and cemento-osseous dysplasia-associated simple bone cysts. Dentomaxillofac Radiol.

[6] Suei Y, Taguchi A, Tanimoto K (2007). Simple bone cyst of the jaws: evaluation of treatment outcome by review of 132 cases. J Oral Maxillofac Surg.

[7] Murad MH, Sultan S, Haffar S, Bazerbachi F (2018). Methodological Quality and Synthesis of Case Series and Case Reports. BMJ Evid Based Med.

[8] Horner K, Forman GH (1988). Atypical simple bone cysts of the jaws. II: A possible association with benign fibro-osseous (cemental) lesions of the jaws. Clin Radiol.

[9] Mahomed F, Altini M, Meer S, Coleman H (2005). Cemento-osseous dysplasia with associated simple bone cysts. J Oral Maxillofac Surg.

[10] Zillo Martini M, Caroli Rocha A, Lemos CA, Abreu Alves F (2010). Fibro-osseous lesions associated with simple bone cysts: three case reports and review of the literature. Minerva Stomatol.

[11] Rollin M, Taihi I (2024). Simple bone cyst within florid cemento-osseous dysplasia: a report of two cases. Cureus.

[12] Rao KA, Shetty SR, Babu SG, Castelino RL (2011). Co-occurence of florid cemento-osseous dysplasia and simple bone cyst: a case report. J Oral Maxillofac Res.

[13] Berberi A (2024). Clinical, CBCT and histological analysis of a florid cemento-osseous dysplasia with co-occurrence of simple bone cyst in the mandible: a case report. J Dent (Shiraz).

[14] Miyauchi M, Ogawa I, Takata T, Ito H, Nikai H, Ijuhin N (1995). Florid cemento-osseous dysplasia with concomitant simple bone cysts: a case in a Japanese woman. J Oral Pathol Med.

[15] Higuchi Y, Nakamura N, Tashiro H (1988). Clinicopathologic study of cemento-osseous dysplasia producing cysts of the mandible : report of four cases. Oral Surg Oral Med Oral Pathol.

[16] Saito Y, Hoshina Y, Nagamine T, Nakajima T, Suzuki M, Hayashi T (1992). Simple bone cyst: a clinical and histopathologic study of fifteen cases. Oral Surg Oral Med Oral Pathol.

[17] Wakasa T, Kawai N, Aiga H, Kishi K (2002). Management of florid cemento-osseous dysplasia of the mandible producing solitary bone cyst: report of a case. J Oral Maxillofac Surg.

[18] Lee YP, Huang BW, Chiang CP, Hwang MJ (2020). Florid cemento-osseous dysplasia with concomitant occurrence of two simple bone cysts in the mandible: case report. J Dent Sci.

[19] Kojima I, Nishioka T, Sakamoto M, Sai Y, Ezoe Y, Iikubo M (2020). Florid cemento-osseous dysplasia-associated simple bone cyst showing marked irregular border and high apparent diffusion coefficient value. Case Rep Dent.

[20] Gumru B, Akkitap MP, Deveci S, Idman E (2021). A retrospective cone beam computed tomography analysis of cemento-osseous dysplasia. J Dent Sci.

[21] Hajjami F, Ouertani H, Brahem H, Mehrez H, Blouza I, Khattech MB (2023). Association of simple bone cyst and cemento-osseous dysplasia: a long-term follow-up. Clin Case Rep.

[22] Yeom HG, Yoon JH (2020). Concomitant cemento-osseous dysplasia and aneurysmal bone cyst of the mandible: a rare case report with literature review. BMC Oral Health.

[23] Kungoane T, Robinson L (2021). Florid cemento-osseous dysplasia with a concurrent glandular odontogenic cyst. Head Neck Pathol.

[24] Harnet JC, Lombardi T, Klewansky P, Rieger J, Tempe MH, Clavert JM (2008). Solitary bone cyst of the jaws: a review of the etiopathogenic hypotheses. J Oral Maxillofac Surg.

[25] Sarmento DJS, Carvalho SHG, Araújo JCWP, Carvalho MV, Silveira ÉJD (2017). Florid cemento-osseous dysplasia and peripheral giant cell granuloma in a patient with neurofibromatosis 1. An Bras Dermatol.

[26] Soluk-Tekkesin M, Sinanoglu A, Selvi F, Karabas HC, Aksakalli N (2022). The importance of clinical and radiological findings for the definitive histopathologic diagnosis of benign fibro-osseous lesions of the jaws: study of 276 Cases. J Stomatol Oral Maxillofacial Surg.

[27] MacDonald-Jankowski DS (2003). Florid cemento-osseous dysplasia: a systematic review. Dentomaxillofacial Radiol.

[28] Fedato Tobias RS, Teodoro AB, Evangelista K, Leite AF, Valladares-Neto J, de Freitas Silva BS (2024). Diagnostic capability of artificial intelligence tools for detecting and classifying odontogenic cysts and tumors: a systematic review and meta-analysis. Oral Surg Oral Med Oral Pathol Oral Radiol.

